# Impact of COVID-19 on telepsychiatry at the service and individual patient level across two UK NHS mental health Trusts

**DOI:** 10.1136/ebmental-2021-300287

**Published:** 2021-09-28

**Authors:** James SW Hong, Rebecca Sheriff, Katharine Smith, Anneka Tomlinson, Fathi Saad, Tanya Smith, Tomas Engelthaler, Peter Phiri, Catherine Henshall, Roger Ede, Mike Denis, Pamina Mitter, Armando D'Agostino, Giancarlo Cerveri, Simona Tomassi, Shanaya Rathod, Nick Broughton, Karl Marlowe, John Geddes, Andrea Cipriani

**Affiliations:** 1 Department of Psychiatry, University of Oxford, Oxford, UK; 2 Oxford Health NHS Foundation Trust, Oxford, Oxfordshire, UK; 3 Akrivia Health, Oxford Centre for Innovation, Oxford, UK; 4 Southern Health NHS Foundation Trust, Southampton, UK; 5 Faculty of Health and Life Sciences, Oxford Brookes University Faculty of Health and Life Sciences, Oxford, UK; 6 Department of Health Sciences, University of Milan, Milano, Lombardia, Italy; 7 Department of Psychiatry and Addiction, ASST Lodi, Lodi, Lombardia, Italy; 8 Psychiatric Unit 1, Azienda ULSS 9 Scaligera, Verona, Veneto, Italy

**Keywords:** adult psychiatry, delirium & cognitive disorders, eating disorders, depression & mood disorders

## Abstract

**Background:**

The effects of COVID-19 on the shift to remote consultations remain to be properly investigated.

**Objective:**

To quantify the extent, nature and clinical impact of the use of telepsychiatry during the COVID-19 pandemic and compare it with the data in the same period of the 2 years before the outbreak.

**Methods:**

We used deidentified electronic health records routinely collected from two UK mental health Foundation Trusts (Oxford Health (OHFT) and Southern Health (SHFT)) between January and September in 2018, 2019 and 2020. We considered three outcomes: (1) service activity, (2) in-person versus remote modalities of consultation and (3) clinical outcomes using Health of the Nation Outcome Scales (HoNOS) data. HoNOS data were collected from two cohorts of patients (cohort 1: patients with ≥1 HoNOS assessment each year in 2018, 2019 and 2020; cohort 2: patients with ≥1 HoNOS assessment each year in 2019 and 2020), and analysed in clusters using superclasses (namely, psychotic, non-psychotic and organic), which are used to assess overall healthcare complexity in the National Health Service. All statistical analyses were done in Python.

**Findings:**

Mental health service activity in 2020 increased in all scheduled community appointments (by 15.4% and 5.6% in OHFT and SHFT, respectively). Remote consultations registered a 3.5-fold to 6-fold increase from February to June 2020 (from 4685 to a peak of 26 245 appointments in OHFT and from 7117 to 24 987 appointments in SHFT), with post-lockdown monthly averages of 23 030 and 22 977 remote appointments/month in OHFT and SHFT, respectively. Video consultations comprised up to one-third of total telepsychiatric services per month from April to September 2020. For patients with dementia, non-attendance rates at in-person appointments were higher than remote appointments (17.2% vs 3.9%). The overall HoNOS cluster value increased only in the organic superclass (clusters 18–21, n=174; p<0.001) from 2019 to 2020, suggesting a specific impact of the COVID-19 pandemic on this population of patients.

**Conclusions and clinical implications:**

The rapid shift to remote service delivery has not reached some groups of patients who may require more tailored management with telepsychiatry.

## Background

Telepsychiatry is the delivery of psychiatric assessments or follow-up interviews from a distance using technologies such as telephone calls, audio and video digital platforms, and healthcare monitoring devices.^1^ After years of protracted efforts to implement digital transformation in the National Health Service (NHS),^2^ the COVID-19 pandemic, and its associated UK-wide lockdown, led to near-overnight adoption of telepsychiatric services in clinical care. This unavoidable transition has undoubtedly ensured continuity of mental healthcare for patients.^3^ However, the impact of this profound shift, in terms of clinical effectiveness and patient satisfaction, and of the pandemic on patients, clinicians and services remains to be quantified.^4^

Rapid reporting of country-specific, high-quality evidence is mandatory to inform relevant stakeholders at a time of unprecedented transformations in healthcare delivery. The wide-ranging impact of telepsychiatry on mental health service use and outcomes of care can be analysed efficiently with the UK’s electronic health records (EHR) infrastructure. The Clinical Record Interactive Search (CRIS) provides an automated platform on which to access secure, deidentified, real-world EHR data and gain clinically meaningful insights into changes at the service and individual patient levels.^5^ Previous studies have assessed the impact of the pandemic on broad population-level mental health outcomes,^6^ but few studies have quantified service-level and patient-level changes with a focus on telepsychiatry. Stewart and colleagues used CRIS to quantify pandemic-related changes in adult community mental health and home treatment teams.^7^ This study was, however, limited to the analysis of data from one Trust only and between 2019 and 2020, making it difficult to differentiate the impact of the COVID-19 pandemic from general trends in the population.

To overcome these shortcomings and increase clinical validity and generalisability of findings, we conducted a study using EHR data from two UK NHS Trusts over a 3-year evaluation period. Our aim was to assess the impact of the COVID-19 pandemic on mental health services by quantifying the extent, nature and patient-level impact of the shift to telepsychiatry.

## Methods

In May 2020, we contacted two NHS Trusts across England, Oxford Health NHS Foundation Trust (OHFT) and Southern Health NHS Foundation Trust (SHFT), which agreed to participate in the study. OHFT provides specialist mental health, physical health and social care services, covering a population of 1.9 million people across Oxfordshire, Buckinghamshire, Swindon, Wiltshire, Bath and North East Somerset. SHFT provides specialist mental health services, learning disability services, social care and integrated community healthcare services covering 1.8 million people in Hampshire, excluding Portsmouth City, with a mixture of urban city, suburbia and rural communities. The two Trusts operated different local EHR systems: CareNotes in OHFT and Servelec Open Rio in SHFT.

Using Structured Query Language (SQL), deidentified mental healthcare data were extracted from local EHR systems by local Data Science Teams (FS, TS, PP, TE) for all patients accessing mental health services at OHFT and SHFT between 1 January and 30 September in 2018, 2019 and 2020 (see online supplemental appendix for data plan, variables and definitions, and services/teams). The same 9-month period across three different years was selected to capture the key moments of the COVID-19 pandemic before and after the first national lockdown on 23 March 2020, and to facilitate high-level serial comparisons across equivalent time frames. The period of analysis was limited to this 9-month period as the focus of the study was on the acute effects of a rapid, system-wide service transformation (ie, 3 months before and 6 months after the national lockdown). The full study protocol is available in the online supplemental appendix. NHS Trust approval was provided by each participating site. Data handling was compliant with NHS Information Governance regulations which include the Data Protection Act. A Patient and Public Involvement representative (RE) was involved in discussions during the planning, analysis and manuscript writing phases of the project.

10.1136/ebmental-2021-300287.supp1 Supplementary data

### HoNOS data

For the analysis of clinical outcomes, we used the HoNOS data (as they are routinely collected in the NHS) and identified two cohorts of patients at OHFT. SHFT could not contribute to this analysis, as the Trust does not readily store cluster-level HoNOS scores in an anonymised format (see below for a description of clusters). It was therefore not feasible to replicate the HoNOS analysis across both sites. HoNOS is a widely used and clinician-reported mental health outcome instrument comprised of 12 items/subscales, which cover symptom severity, functioning, social and environmental measures.^8^ The HoNOS score is then combined with additional risk-based scales to group patients into ‘clusters’ using the Mental Health Clustering Tool.^9^ The 21 clusters are divided into three superclasses (psychosis, non-psychosis and organic), which are a measure of combined symptomatic-functional-social-environmental severity.^9^ Within each superclass, the cluster value increases along an ordinal scale, reflecting increasing healthcare complexity with correspondingly higher costs for the NHS.^10^

Cohort 1 included patients with at least one HoNOS assessment each year during the index period (ie, 2018, 2019 and 2020); by contrast, cohort 2 included patients with at least one HoNOS assessment during the index period only in 2019 and 2020. The HoNOS scores were linked to the data from clinical visits. If the date of the HoNOS assessment did not match the date of the visit as registered in the system, we took the first attended diary appointment date within 5 days prior to the HoNOS date. If a patient had more than one HoNOS assessment in a given year, the most recent assessment was included in the analysis. A focus group with senior clinical staff at OHFT was carried out to agree on the cluster data analysis and the interpretation of the results. For each cohort, we identified patients who did and did not move to a different superclass in the years considered. Changes in HoNOS scores and clusters were analysed within the same superclass. The Friedman test was used to test for statistical significance in three or more dependent samples. Where appropriate, post hoc analysis for differences between pairs of years (eg, 2018–2019, 2019–2020) was conducted using the Wilcoxon signed-rank test with Bonferroni correction. Where there were only two dependent samples, the Wilcoxon signed-rank test was used. An alpha level of 0.001 was preferred in the interpretation of statistical test results as recommended and used in real-world observational studies of health services.^11 12^ Statistical analyses were performed in Python.

## Findings

### Impact on service delivery

There were 204 504 registered mental health patients in OHFT and 166 702 in SHFT (table 1). Of note, Child and Adolescent Mental Health Service data were not available for SHFT. However, most service activity variables, including referral, discharge and inpatient measures, were common to the two Trusts (table 1; online supplemental appendix, Sections 2 and 5). From 2019 to 2020, there was a reduction in measures of turnover, such as referrals and discharges, in both SHFT and OHFT (table 1). In OHFT, the 2019-to-2020 change in these measures reversed the increasing trend in referral and discharge numbers observed from 2018 to 2019, while SHFT experienced yearly decrease in referrals and discharges from 2018 to 2020 (table 1). Similar yearly trends were found in measures of inpatient service turnover at OHFT, while yearly increases in these measures were observed at SHFT (online supplemental appendix, table 8A). Despite these differences, both Trusts consistently reported a large decrease in referrals per month from March to April 2020 (online supplemental appendix, table 6D and figure 6H) and a decrease in number of distinct inpatients in March 2020 (online supplemental appendix, table 8C and figure 8A). However, monthly referral activity returned to pre-lockdown levels by June/July 2020 (online supplemental appendix, table 6D and 6H).

**Table 1:** Aggregate service measures from 2018 to 2020

	Oxford	Southern
Overall populations served (2020)	1.9 million	1.8 million
Registered in mental health services	204 504	166 702
Open referrals		
2018	66 932	60 076
2019	75 703	57 804
2020	74 307	52 164
New referrals	40 899	
2018	46 818	56 498
2019	42 642	53 863
2020		48 389
New patients with an accepted referral	27 944	
2018	30 216	39 555
2019	26 522	37 865
2020		33 739
Discharges		
2018	39 188	39 710
2019	45 130	39 395
2020	44 202	35 523
Scheduled appointments		
2018	244 216	337 946
2019	253 760	337 893
2020	292 942	356 909

The total number of scheduled appointments increased in 2020 (table 1). In OHFT, there was a 15.4% overall increase in scheduled appointments from 2019 to 2020, compared with a 3.9% increase from 2018 to 2019 (table 1). In SHFT, there was a 5.6% increase in scheduled appointments from 2019 to 2020, compared with a stable number of total appointments from 2018 to 2019 (table 1).

### Remote consultations

**Figure 1:**
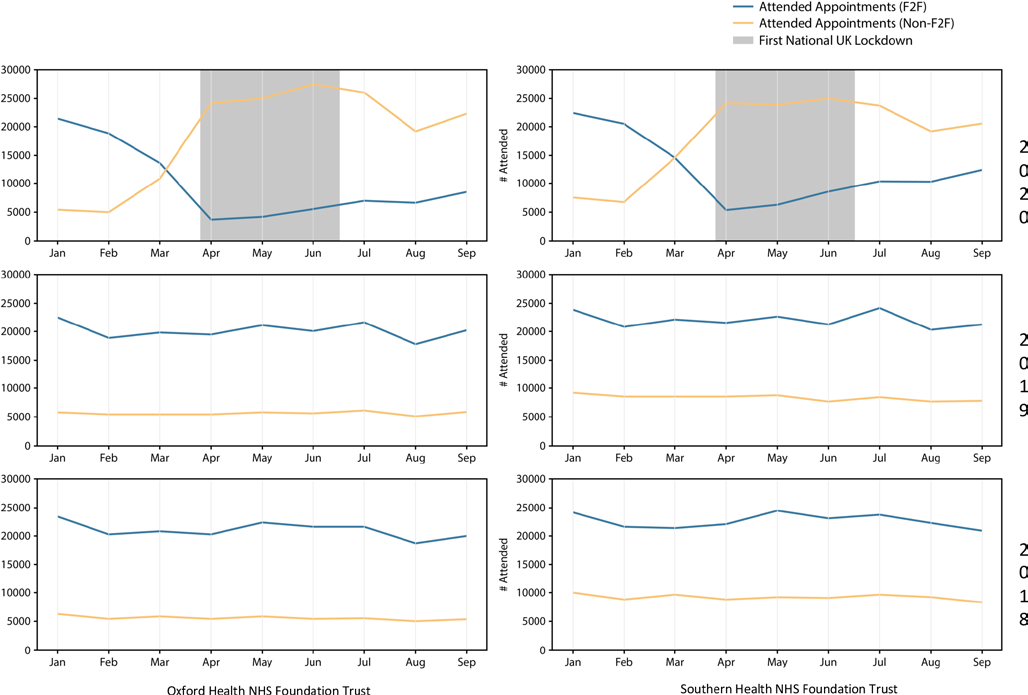
Attended face-to-face (F2F) and non-face-to-face (Non-F2F) appointments.

**Figure 2:**
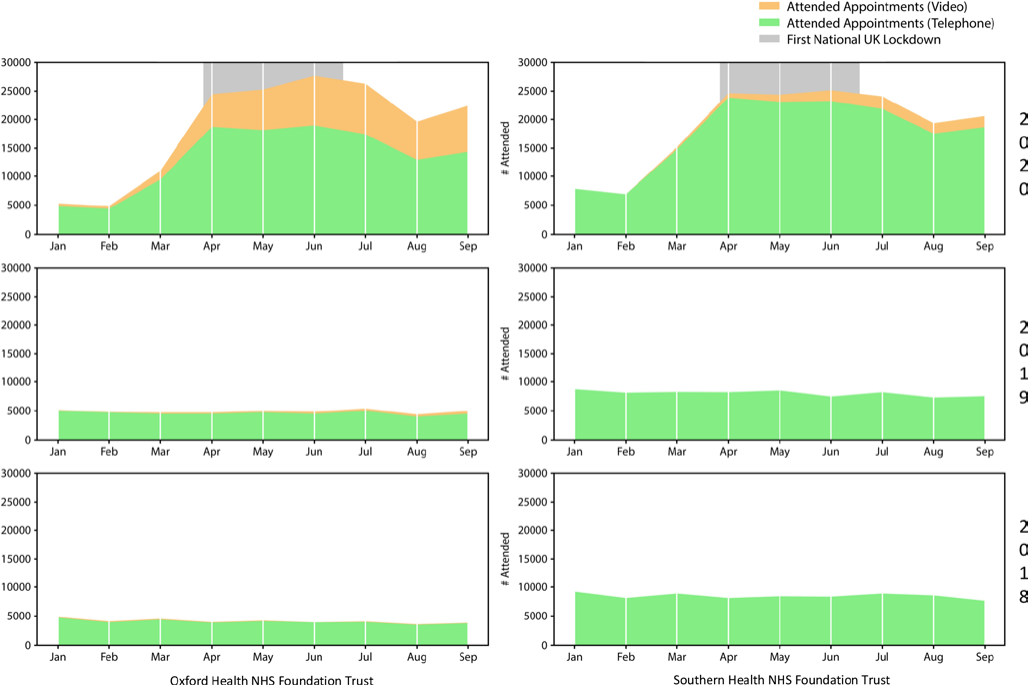
Attended appointments by telephone and video.

**Figure 3:**
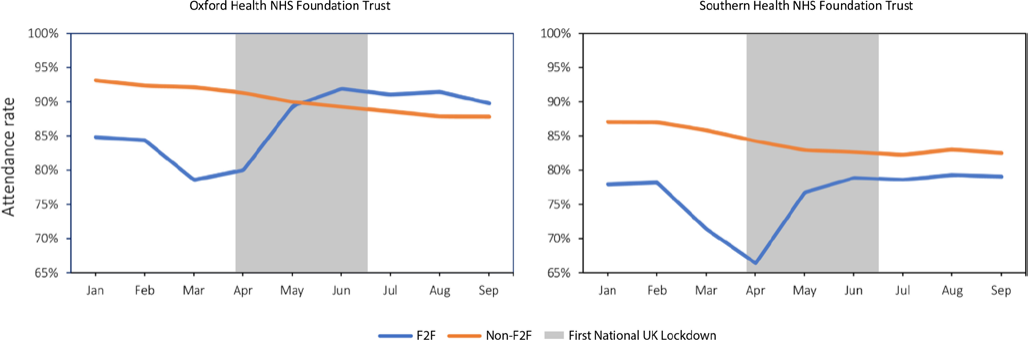
Attendance rate of face-to-face (F2F) and non-face-to-face (Non-F2F) appointments in 2020.

The number of non-face-to-face (remote) appointments—comprising video and telephone consultations—increased in both Trusts during the pandemic, going from 4685 to a peak of 26 245 appointments in OHFT and from 7117 to 24 987 appointments in SHFT, in February to June 2020. The monthly averages from April to September 2020 were 23 030 and 22 977 remote appointments/month in OHFT and SHFT, respectively (figure 1). At OHFT video consultations contributed to an average of 30.5% of the total telepsychiatric services per month from April to September 2020, while, prior to the pandemic, they comprised less than 10% (9.1% in February 2020) (figure 2). SHFT registered a smaller proportion of video consultations out of the total number of telepsychiatry activities (monthly average 7.6% from April to September 2020), but it is worth noting that less than 0.2% of all remote consultations were done by video before March 2020 (figure 2). The baseline attendance rate in February 2020, regardless of modality, was lower in SHFT (78.2% for in-person and 87.1% for remote) than OHFT (84.4% and 92.4%, respectively) (figure 3; online supplemental appendix, table 6E). Month-by-month analysis showed that attendance rate to remote visits decreased between January and September 2020 in both Trusts, and at OHFT, it became lower than the attendance rate of in-person appointments from May 2020 onwards (figure 3; online supplemental appendix, table 6E). After the lockdown, no-show rates increased for remote appointments at OHFT (4.6% in February to 8.2% in September 2020) and decreased for in-person appointments at OHFT (from 6.1% to 4.8%) and SHFT (from 9.6% to 7.0%) (online supplemental appendix, figure 6J). No-show rates at remote appointments at SHFT remained largely stable pre-to-post lockdown, with a rate of 11.6% in both February and September 2020 (online supplemental appendix, figure 6J).

The analysis of diagnostic group-level data (based on International Statistical Classification of Diseases and Related Health Problems, version 10 codes, or treatment team) showed that the ratio of remote to in-person appointments increased from 2019 to 2020 in all diagnostic groups (online supplemental appendix, figure 6C, G). The greatest change was for Eating Disorders (from 0.12 to 1.51 and 0.10 to 2.53 at OHFT and SHFT, respectively; online supplemental appendix, table 6B, C), with other groups, such as mental retardation (2.61), developmental disorders (2.20), dementia (2.11) and conduct disorders (2.05) to follow (online supplemental appendix, table 6B). For patients with dementia at OHFT, non-attendance rates at in-person appointments were fourfold higher than remote appointments (17.2% vs 3.9%) (online supplemental appendix, Table 6F).

### Analysis of HoNOS data

Only OHFT reported HoNOS data. Cohort 1 (2018, 2019, 2020) comprised 998 patients (online supplemental appendix, table 7A). Of them, 792 patients (79.4%) stayed within the same superclass: 284 (35.9%) non-psychotic, 174 (22.0%) organic and 332 (41.9%) psychotic (2 remaining patients were in the ‘variance cluster’, ie, in need of mental healthcare, but not classified into any superclass) (online supplemental appendix, table 7B). Analysing the change of clusters within the same superclass, only the organic superclass reported a statistically significant difference in the cluster value between 2018 and 2020 (p<0.001) (online supplemental appendix, table 7L). A post hoc pairwise test showed that the significant increase happened between 2019 and 2020, but not between 2018 and 2019 (online supplemental appendix, table 7N). The results of the cluster analysis were supported by the analysis of HoNOS total scores within the same superclass (online supplemental appendix, tables 7E, 7F, 7H).

Cohort 2 (2019, 2020) included 2712 patients, of whom 2318 (85.5%) stayed within the same superclass from 2019 to 2020: 909 patients (39.2%) were non-psychotic, 687 (29.6%) organic, 720 (31.1%) psychotic and 2 (0.1%) in the variance cluster (online supplemental appendix, tables 7O, P). The analysis of clusters within the same superclass showed increased complexity in the organic superclass (p<0.001) (online supplemental appendix, table 7Z). In these patients, the HoNOS total score worsened significantly (p<0.001), while the non-psychotic and psychotic superclass showed an improvement in total HoNOS score (online supplemental appendix, Table 7U). The complete description of HoNOS results is available in the online supplemental appendix, Section 7.

## Discussion

Our study of two large NHS mental health Trusts demonstrated a rapid shift to remote service delivery as a result of the COVID-19 pandemic and associated national lockdown. These findings are consistent with other published reports.^7 13–15^ However, this is the first study assessing the clinical impact on mental health services and patients. Taken together, our study suggests that remote service activity increased markedly, with considerable use of video consultations, but this did not automatically translate to more patients attending the scheduled appointments. Notably, our findings on non-attendance differ with the experience of mental healthcare providers in the USA who have reported reductions in tele-mental health no-show rates after rapid virtualisation of services.^16 17^ In contrast, no-show rates at remote consultations increased in OHFT and remained stable in SHFT after lockdown-related service changes. We could not directly compare associated transatlantic clinical outcomes due to a dearth of such analyses in the literature. However, our study suggests that the impact on service activity is health system, region and diagnosis specific. Associated changes in clinical outcomes may also partly reflect such differences, but this currently remains a hypothesis to be tested. Importantly, our analysis of clinical outcomes shows that they can vary according to the broad diagnostic ‘superclasses’ as defined in the UK mental healthcare system.

In terms of clinical outcomes, during the pandemic, patients belonging to the organic superclass significantly increased in healthcare complexity in comparison with previous years. While ICD-10 diagnoses and HoNOS clusters are not exactly the same,^9^ there is up to 76% overlap between the two for inpatients with a diagnosis of dementia and organic disorders.^18^ HoNOS scores can be a significant resource to gain insights into mental illness and treatment effectiveness.^19^ These findings are consistent with reports of pandemic-related worsening of behavioural and psychological symptoms in people with dementia,^20^ and can have implications for patients, carers and mental health services at different levels. While the remote attendance rates of patient with dementia were high, the effectiveness and usefulness of these consultations have not been established. A recent review suggests that remote management of dementia could achieve a level of diagnostic accuracy and patient and caregiver satisfaction comparable to in-person consultations.^21^ However, factors such as reduced cognitive function, confusion and sensory impairments may reduce the quality of teleconsultations for these patients as they may find it difficult to engage with their healthcare clinician using a virtual format.^20^ Literacy, availability and familiarity with the internet may also be an issue. For instance, in the UK in 2019, only 47% of adults above 75 years of age were considered recent internet users, compared with virtually all (99%) adults aged 16–44 years.^22^ Patient difficulties with using technology for telepsychiatry have been reported in both the 55–64 and 65–74 year old age groups.^17^

The eating disorders (ED) group had the greatest relative pre-to-post increase in the use of telepsychiatry. Evidence suggests that the psychological well-being of individuals with ED has been particularly impacted by the pandemic.^23^ One ED-focused service evaluation study showed unexpected benefits of teleconferencing, including increased participation of patients in therapeutic groups and enhanced interdisciplinary communication between staff, but stated that significant practical challenges remained in the implementation of telepsychiatry for people with ED.^24^

Questions remain about the wider applicability of telepsychiatry across the whole spectrum of mental health disorders. For personality disorders, schizophrenia and substance misuse diagnostic groups, in our study the ratio of remote versus in-person appointments was less than 1, indicating greater difficulty of remotely managing patients characterised by a more disruptive behavioural component. Further work remains to be done in better understanding how telepsychiatry services could be improved, and for whom and in what situations different remote modalities are most appropriate. This is important, as our study suggests, that the rapid shift to remote service delivery has not reached some groups of patients who may require more tailored management with telepsychiatry. Specific groups of patients may require more intensive and/or nuanced management in the post-pandemic era of digital psychiatry. Hybrid models of care, combining digital psychiatry with face-to-face assessment and care coordinators to support patients in overcoming practical problems, may be an effective way of managing highly complex groups of patients with a more personalised and blended approach. The quality, effectiveness and uptake of telepsychiatry can be enhanced by training clinicians^25^ and patients.^26^ There is a need for evidence-based, targeted telepsychiatric training programmes to enhance clinical efficacy and health outcomes in patients with mental health disorders. Training will be especially important not only in terms of assessment of patients via video or telephone consultations but also in terms of remote monitoring of symptoms^27^ and delivery of treatment modules.^28^

The strengths of our study include the size of the dataset, comparison across two large NHS mental health Trusts in England and the use of patient-level data to characterise the impact of the pandemic, using data analysed over 3 years to conduct serial comparisons. However, the HoNOS analysis was not replicated at SHFT as the Trust does not readily store cluster-level HoNOS data in an anonymised format; hence, the patient-level outcomes are limited to OHFT. Moreover, the months considered were limited to January–September, meaning some significant events, such as the second national lockdown (5 November 2020), were excluded. Additional studies should be conducted to examine the long-term patterns in telepsychiatry use and the impact on clinical outcomes beyond the pandemic-associated acute service transformation. Furthermore, the findings cannot immediately be generalised to populations or healthcare settings outside of the Trusts. Patients seen in secondary/specialist mental health settings have distinct, often more complex, needs compared with those who are treated in primary care mental health settings.^29^ Future studies should aim to quantify these differences, adjust for individual and regional socioeconomic factors, and consider Trust-specific differences in the structure of teams and delivery of mental health services.

This work is primarily a descriptive study of service changes. Patient outcomes and various dimensions of the quality of telepsychiatric services, such as effectiveness, utility and acceptability, cannot be definitively correlated. While we have demonstrated that it is feasible to use HoNOS data to assess patient outcomes and generate clinically meaningful insights,^8 30^ the HoNOS has been shown to have moderate inter-rater reliability and limited validity in relation to patient-reported symptomatology.^31^

Our work reveals challenges in conducting a study using EHR from different mental health Trusts, each one with their own data infrastructure. OHFT and SHFT had different local EHR systems with some, although relatively minor, variations in definitions of variables, and the data infrastructure. A shared data plan was developed in the protocol and aimed at ensuring overall consistency in extracting variables of interest, their descriptions and optimising workflow. The collaboration required frequent, often separate discussions between individual data teams to ensure that analyses represented the same underlying variables, produced comparable visualisations and made any amendments to reflect theoretical iterations of the project. As EHR studies may generate important data-driven insights during the COVID-19 pandemic and beyond, more efficient and integrated data infrastructures, such as the development of a federated database, are likely to play an increasingly important role in the future of mental health research and service improvement processes.^32^

## Data Availability

Data may be obtained from a third party and are not publicly available.
