# Supplementary material for: Impact of COVID-19 on telepsychiatry at the service and individual patient level across two UK NHS mental health Trusts
Source: Evid Based Ment Health. 2021 Sep 28:ebmental-2021-300287. doi: 10.1136/ebmental-2021-300287 (PMC8483920; doi:10.1136/ebmental-2021-300287)
Supplement: Supplementary file 1 [file ebmental-2021-300287supp001.pdf]

Appendix - Impact of COVID-19 on telepsychiatry at the service and individual patient level (Hong et al., 2021)

Table of Contents

Section 1: Study protocol. Evaluation of telepsychiatry: a feasibility study across the lifespan within the NHS 2

Section 2: Key variable definitions 5

Section 3: Contact/Consultation types: F2F (face-to-face/in-person) and Non F2F (non-face-to-face/remote) 7

Section 4: Mental health services inclusion and exclusion 8

Section 5: Demographic data 9

Section 5A: Southern Health NHS Foundation Trust (SHFT) demographic data 9

Section 5B: Oxford Health NHS Foundation Trust (OHFT) demographic data 11

Section 6: Community mental health services 12

Section 7: HoNOS and Mental Health Care cluster analyses 25

Section 7.1: Cohort 1 Analyses 25

Section 7.1A: HoNOS Scores 26

Section 7.1B: Clusters 29

Section 7.2: Cohort 2 Analyses 31

Section 7.2A: HoNOS Scores 32

Section 7.2B: Clusters 35

Section 8: Inpatient service data 38

References 40

Hong JSW, *et al. Evid Based Ment Health* 2021;0:1–6. doi: 10.1136/ebmental-2021-300287

Section 1: Study protocol. Evaluation of telepsychiatry: a feasibility study across the lifespan within the NHS

The following protocol was designed and submitted to Oxford Health NHS Foundation Trust (OHFT) and Southern Health NHS Foundation Trust (SHFT) committees prior to study initiation.

Background and rationale

Mental health and wellbeing are a major public health concern during the COVID-19 pandemic (Rathod et al., 2020) and higher prevalence of suicidal ideation and lower prevalence of wellbeing reported in the general public (Phiri et al., 2021). Health anxiety, as well as the impact of social distancing, and the accompanying economic disruption are likely to have a wide and far reaching impact on the mental health and wellbeing of the population, both now and into the future (Chau et al., 2021).

As well as the health impact on the general population, there is likely to be increased demand from existing service users, who are particularly vulnerable at this difficult time. In addition, the needs of the general public are likely to prompt increased numbers of referrals for assessment by mental health services. However, the need for health services to follow social distancing measures to reduce spread of COVID-19 means that there have been changes in the way health services have been working. Face-face contact with patients has been minimised and health staff have been encouraged to offer consultations remotely. However, early indications are that the uptake of different types of telehealth consultations (telephone or video-based) are patchy, with significantly more video consultation in Child and Adolescent services and very few in adult and older age services (Oxford Health).

Whilst this may be a new approach for many clinicians and patients, telepsychiatry is a well-recognised sub-discipline of telemedicine and includes psychiatric assessments or follow-up interviews conducted using telephone calls, audio and video digital platforms. It has been used since the 1950s and, particularly in the USA and Australia has been subject to evaluations with promising outcomes and clear guidelines.(APA 2021, https://www.psychiatry.org/psychiatrists/practice/telepsychiatry/toolkit/history-of-telepsychiatry). The evidence suggests that it is effective and well accepted across a range of age groups and cultural backgrounds. It may also be able to offer additional benefits, particularly when combined with other digital technologies such as online platforms and apps for recording and tracking of symptoms and treatment strategies. However, it is unclear to what extent UK clinicians are aware of this and are utilising the research and guidelines related to telepsychiatry during this current response to the COVID-19 pandemic.

The current crisis represents both a challenge and an opportunity for mental health services. Whereas there is evidence that telepsychiatry can be used to offer effective care for mental health problems (https://oxfordhealthbrc.nihr.ac.uk/our-work/oxppl/table-5-digital-technologies-and-

telepsychiatry/), there is a dearth of evidence regarding incorporation of telepsychiatry into routine mental health services in the context of a disaster. Therefore, the onus is on mental health services to optimise the use of telemedicine using the best available evidence and also to conduct meaningful research into its implementation at a time of crisis.

Even prior to the COVID-19 pandemic mental health services were already looking at service transformation to increase the use of digital technologies for consultations. The COVID-19 pandemic and social distancing has made evaluating such a transformation even more urgent. The results of such an evaluation will be essential both during and in the aftermath of the pandemic.

Aims

We aim to conduct a feasibility study to evaluate the uptake, acceptability (including barriers to use), clinical utility and overall service impact of telepsychiatry during the COVID-19 pandemic within the NHS. This will lay the groundwork for larger-scale studies, in due course, evaluating the impact of interventions such as remote training, introduction of apps (to support teleconsultations), and/or use of clinical-decision making tools in teleconsultations.

Objectives

1) To evaluate the *uptake* of teleconsultations, using routine clinical data to analyse numbers of telephone, digital and face to face contacts
2) To evaluate the *acceptability* of teleconsultations, using online feedback from clinicians, service-users and carers, as to how these consultations are experienced. Barriers to use, such as lack of confidence in using technology, or lack of access to the technology itself will be assessed, with options for written or telephone feedback if easier
3) To evaluate the *utility* of teleconsultations, using routine clinical data (such as DNAs, consultation time, amount/quality/rapidity of documentation) alongside online questionnaire data from service-users and clinicians (perceived communication and rapport)
4) To evaluate the *effectiveness* of teleconsultations using routine clinical data (such as prescriptions, hospitalisation) alongside online questionnaires from clinicians (PHQ/global state) and service users (self-report measures of global state)
5) Overall impact on the service- qualitative data on overall effects from clinicians, service users and carers.
6) To develop an intervention to improve telepsychiatry within the NHS
7) To develop a protocol for a trial to evaluate the intervention to implement telepsychiatry in the NHS

Approach

It will be important to frame the evaluation within the context of the current, and growing, research evidence base relating to telepsychiatry initiatives and the application of this evidence as is reflected in existing training and guidance materials available within the Trust.

Method

In Phase 1 we will collect and analyse data, using routine clinical data and self-report questionnaires developed specifically for this study (as outlined in objectives 1-5). For each of the above outcomes we will analyse by service factors (e.g. types of service), clinician factors (e.g. discipline) and service user factors (e.g. degree of disability, diagnosis, sex, age), which will be available from routine clinical data. Services to be included in this study will be agreed with the Trust.

In Phase 2 we will develop an intervention aimed at improving the uptake, acceptability, utility and/or effectiveness of telepsychiatry within NHS services. Most likely it will consist of a package of information and training. The content, format and delivery of which will be decided in consultation with service users, clinicians and managers and using the best available evidence in the literature. Focus groups with clinicians and patients, together with other feedback, will be used to develop this intervention with expert input.

In Phase 3 we will develop a protocol for a trial to evaluate the developed intervention, most likely using a stepped wedge design.

Participants

Patients, carers and clinicians in NHS mental health services (including children and adolescents, adults, older adults, pharmacies and care homes).

Out of Scope

This study will exclude any form of evaluation of types of digital platforms being used in the Trust.

Expected impact

This is a feasibility study to evaluate the current use of telepsychiatry and barriers to its use, in the context of COVID-19. The information would be used to design an intervention study to improve patient care (nationally and possibly internationally) in the context of the current pandemic and telepsychiatry beyond this.

Section 2: Key variable definitions

**Service activity variables** **(between 1st Jan and 1st Sept 2018, 2019, 2020)**	**Definitions** **(All variables exclude test (dummy) patients/referrals)**
**Total number of patients registered in mental health services**	Number of patients registered to MH - number in patient table (client demographics). Will include deceased.
**Total number of distinct patients seen during the study period**	Patients who have had an open referral during this period - counted only once. This will include deceased for those which have had an open referral and subsequently become deceased within the same period.
**Total number of patients with an open referral**	Extracted in accordance to how the Trust report Nationally - Open referral is defined as a referral accepted date with no end date or where the end date occurred within the period. **Exclusions**: rejected referrals. This is a **distinct count of patients** with an open referral within the period
**Total number of open referrals**	Extracted in accordance to how the Trust report Nationally - Open referral is defined as a referral accepted date with no end date or where the end date occurred within the period. **Exclusions**: rejected referrals. This counts **number of referrals within the period** (more than one per patient is possible)
**Total number of new patients with an accepted referral**	Extracted in accordance to how the Trust report Nationally - Accepted referral is defined as a referral accepted date within the period. **Exclusions**: rejected referrals. This is a **distinct count of patients** with an accepted referral within the period. The patient could have been subsequently discharged within the same period or had multiple referrals accepted within the period, they will only be counted once.
**Total number of new referrals**	Extracted in accordance to how the Trust report Nationally - Accepted referral is defined as a referral accepted date within the period. **Exclusions**: rejected referrals. This is a **count of number of** accepted referrals within the period. More than one per patient is possible.
**Number of patients discharged**	Discharge is where the referral has a discharge date within the period. Distinct count of patients. If multiple referrals and discharged within the same period then only one is counted.

**Number of Discharges**	Discharge is where the referral has a discharge date within the period. **Excluding** test patients. Count of number of discharges within the period. If a patient has multiple discharges within the same period then all discharges are counted
**Number of patient deaths**	Count of patient deaths which fall within the periods
Inpatient activity variables	***Definitions***
**Number of Inpatients (number of occupied beds per day)**	Number of occupied beds per day - If a patient has a bed for 5 days then 7 days within the same period then this will count as 12
**Number of inpatients (were in hospital at least once during the period)**	Number of distinct patient with a hospital stay
**Number of new inpatients (admissions)**	Number of admission start dates for inpatients within the study period. Can be multiple per patient if more then one admission occurs within the period.
**Number of discharged inpatients**	Number of discharge dates for inpatients within the study period. Can be multiple per patient if more then one discharge occurs within the period.

Section 3: Contact/Consultation types: F2F (face-to-face/in-person) and Non F2F (non-face-to-face/remote)

Contact Types	F2F / Non F2F	Contact Type Group
NULL		
Digital Consultations/Telemedicine	Non F2F	Telemedicine
Email	Non F2F	Email
Face to face communication	F2F	Face to Face
Short Message Service (SMS) – Text Messaging	Non F2F	SMS
Talk Type for a Person Unable to Speak	Non F2F	Talk Type
Telemedicine	Non F2F	Telemedicine
Telemedicine Web Camera	Non F2F	Telemedicine
Telephone	Non F2F	Telephone

Section 4: Mental health services inclusion and exclusion

Services	Include	Exclude
CAMHS		Exclude Learning Disabilities (LD), Eating Disorders (ED), Forensic, IAPT and psychological therapies, Psychiatric liaison services, Rehabilitation Services, Tertiary, Neuro psychiatry
Adults	Perinatal, Complex needs	Learning Disabilities (LD), Eating Disorders (ED), Forensic, IAPT and psychological therapies, Psychiatric liaison services, Rehabilitation Services, Tertiary, Home Treatment Teams/Crisis services, Neuro psychiatry
Older Adults		Learning Disabilities (LD), Eating Disorders (ED), Forensic, IAPT and psychological therapies, Psychiatric liaison services, Rehabilitation Services, Tertiary, Home Treatment Teams/Crisis services, Neuro psychiatry, memory clinics?
Eating Disorders	Adults and CAMHS ED	
Forensic Mental Health	Adults and CAMHS Forensic	
Learning Disabilities	Adults and CAMHS LD	
EIP	Adult and CAMHS EIP	
Memory Clinics	Memory Assessment Services	
Liaison	Liaison / A&E services, independent of age of the patient - including off-site emergency clinics that may have been set up to carry out assessments that would normally be expected to take place in A&E	Specific perinatal
HTT & Crisis Response	Home treatment / crisis services - independent of age of the patient	

Section 5: Demographic data

Section 5A: Southern Health NHS Foundation Trust (SHFT) demographic data

Tables 5Ai-iv below show: SHFT service data by age, gender, ethnicity, and team-level % figures. Calculations were done using initial absolute number of *numbers of accepted referrals*, for all services including inpatient and community. Note that a single patient can have multiple referrals.
Percentages in the table below were calculated by dividing the total number of accepted referrals for the respective year.

Table 5Ai. SHFT demographic data: age.

Year	Age (0-11)	Age (12-13)	Age (14-15)	Age (16-17)	Age (18-64)	Age (65+)
2018	0.04%	0.19%	0.67%	1.98%	65.98%	31.15%
2019	0.04%	0.09%	0.48%	1.65%	66.83%	30.92%
2020	0.04%	0.08%	0.24%	1.38%	67.28%	30.99%
Average across 2018-2020		0.12%	0.46%	1.67%	66.70%	31.02%

Table 5Aii. SHFT demographic data: gender.

Year	Gender (Female)	Gender (Male)	Gender (Not Stated)	Gender (Other)
2018	55.00%	44.93%	0.06%	0.02%
2019	57.27%	42.69%	0.04%	0.01%
2020	58.60%	41.36%	0.03%	0.01%
Average across 2018-2020	56.96%	42.99%	0.04%	0.01%

Table 5Aiii. SHFT demographic data: ethnicity.

Year	Ethnicity (Afro-Caribbean)	Ethnicity (Asian)	Ethnicity (British)	Ethnicity (Chinese)	Ethnicity (Not Stated)	Ethnicity (Other)
2018	0.25%	0.43%	73.26%	0.14%	20.77%	5.13%
2019	0.23%	0.44%	74.57%	0.15%	19.21%	5.41%
2020	0.28%	0.42%	74.76%	0.18%	18.60%	5.78%
Average across 2018-2020	0.25%	0.43%	74.20%	0.16%	19.53%	5.44%

Section 5B: Oxford Health NHS Foundation Trust (OHFT) demographic data

Year	Age (0-11)	Age (12-13)	Age (14-15)	Age (16-17)	Age (18-24)	Age (25-34)	Age (35-44)	Age (45-55)	Age (56-64)	Age (65-74)	Age (75+)	Age (Other)
2018	7.89%	5.31%	6.23%	8.65%	20.55%	11.41%	8.79%	8.61%	5.42%	4.05%	13.06%	0.01%
2019	11.36%	5.73%	7.36%	9.77%	16.68%	10.89%	8.57%	8.38%	5.01%	4.01%	12.23%	0.01%
2020	13.57%	6.22%	8.53%	10.49%	13.17%	11.05%	8.53%	8.15%	4.89%	3.94%	11.47%	0.00%
Average across 2018-2020	10.94%	5.75%	7.37%	9.64%	16.80%	11.12%	8.63%	8.38%	5.11%	4.00%	12.26%	0.01%

Table 5Bi. OHFT demographic data: age.

Year	Gender (Female)	Gender (Male)	Gender (Other)
2018	53.26%	46.68%	0.06%
2019	52.90%	47.04%	0.06%
2020	53.63%	46.32%	0.05%
Average across 2018-2020	53.26%	46.68%	0.06%

Table 5Bii. OHFT demographic data: gender.

Year	Ethnicity-African	Ethnicity-Any other Asian background	Ethnicity-Any other Black background	Ethnicity-Any other ethnic group	Ethnicity-Any other mixed background	Ethnicity-Any other White background	Ethnicity-British	Ethnicity-Irish	Ethnicity-Not Known	Ethnicity-Not stated	Ethnicity-White and Asian	Ethnicity-White and Black African	Ethnicity-White and Black Caribbean
2018	0.42%	2.35%	0.81%	0.68%	1.82%	3.20%	61.10%	0.53%	26.33%	1.07%	0.53%	0.34%	0.83%
2019	0.40%	2.35%	0.84%	0.66%	3.12%	2.97%	59.63%	0.46%	26.68%	1.12%	0.57%	0.35%	0.85%
2020	0.40%	2.29%	0.81%	0.99%	3.08%	3.14%	58.18%	0.43%	27.65%	1.12%	0.61%	0.38%	0.92%
Average across 2018-2020	0.41%	2.33%	0.82%	0.77%	2.67%	3.10%	59.64%	0.47%	26.89%	1.10%	0.57%	0.36%	0.87%

Table 5Biii. OHFT demographic data: ethnicity.

Section 6: Community mental health services

Community Mental Health Appointments				
	Aggregate No.	Oxford	Southern	Southern:Oxford ratio in each year
**Scheduled F2F and Non F2F Appointments**				
2018		244,216	337,946	1.38
2019		253,758	337,893	1.33
2020		292,864	356,909	1.22
**Attended F2F and Non F2F Appointments**				
2018		207,781	272,392	1.31
2019		216,617	271,076	1.25
2020		258,265	288,672	1.12

Table 6A: Scheduled and attended F2F (face-to-face/in-person) and Non F2F (non-face-to-face/remote) appointments in community mental health services in Oxford Health NHS Foundation Trust (OHFT) and Southern Health NHS Foundation Trust (SHFT) in 2018, 2019, and 2020.

Figure 6A. Total CMHT activity in OHFT, in terms of F2F plus NF2F appointments, from 2019 to 2020.
X-axis: ICD-10 diagnostic group and year. Y-axis: number of appointments. ICD-10: International Classification of Diseases, 10th edition.

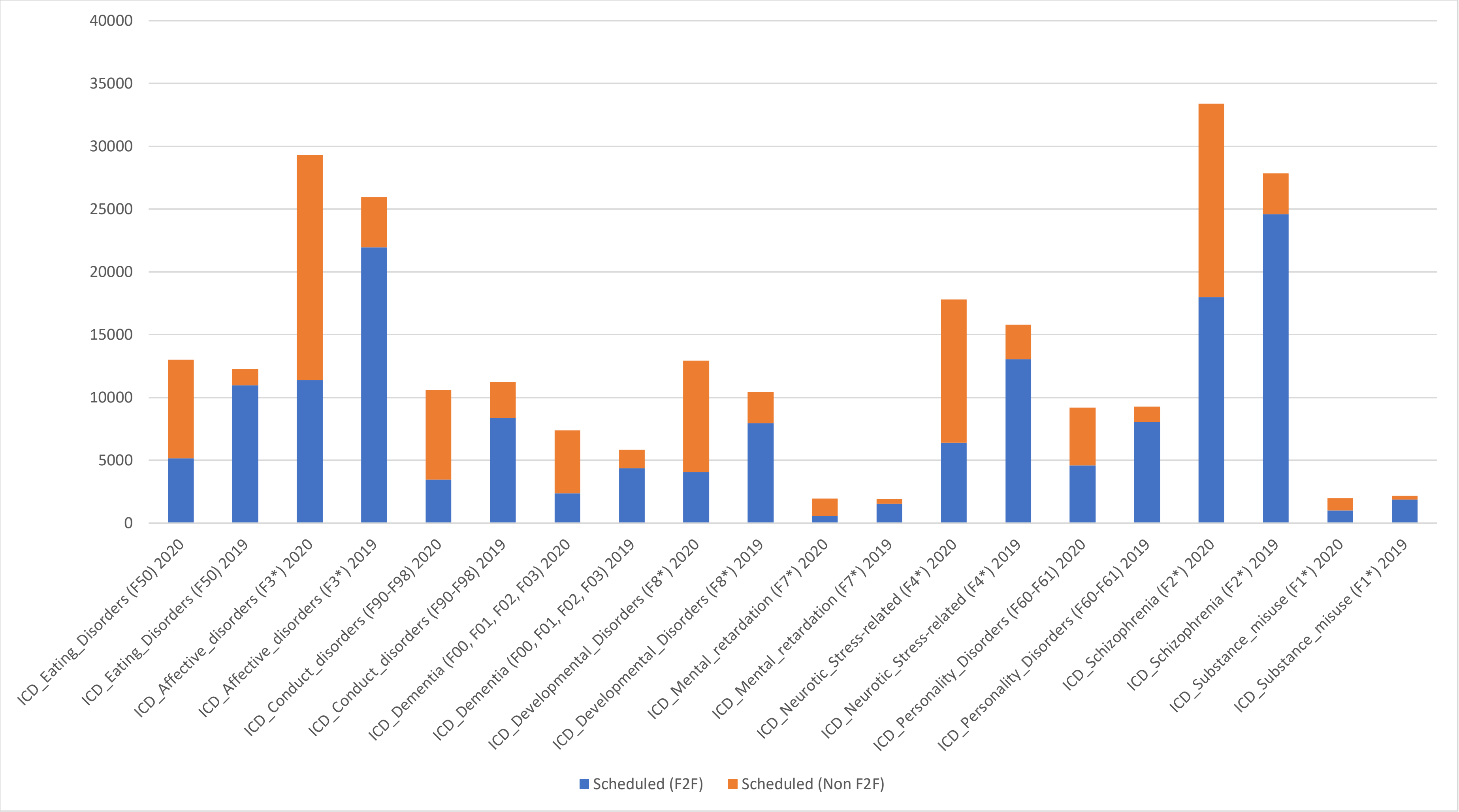

Figure 6B. Total CMHT activity in OHFT, in terms of F2F plus NF2F appointments, from 2019 to 2020, for ICD-10 'Other' Diagnostic group (any diagnostic group not in Figure 6a) and for *all* diagnostic groups combined. Y-axis: number of appointments.

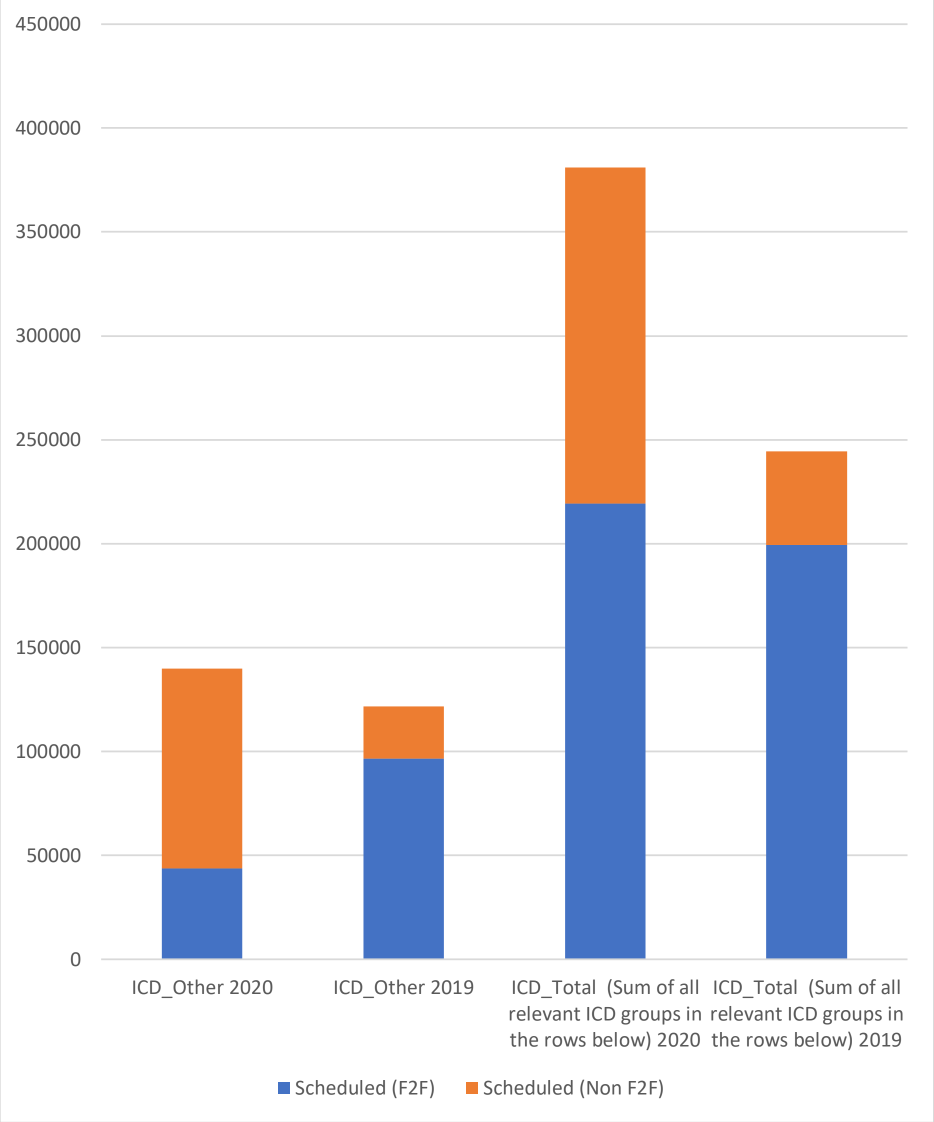

Table 6B: Ratio of scheduled NF2F versus F2F appointments by ICD-10 diagnostic group, in 2019 and 2020, in OHFT.

ICD-10 Diagnostic Group	Ratio Scheduled NF2F to F2F 2019	Ratio Scheduled NF2F to F2F 2020
ICD_Eating_Disorders (F50)	0.12	1.51
ICD_Affective_disorders (F3*)	0.18	1.58
ICD_Conduct_disorders (F90-F98)	0.34	2.05
ICD_Dementia (F00, F01, F02, F03)	0.34	2.11
ICD_Developmental_Disorders (F8*)	0.31	2.20
ICD_Mental_retardation (F7*)	0.24	2.61
ICD_Neurotic_Stress-related (F4*)	0.21	1.79
ICD_Personality_Disorders (F60-F61)	0.15	1.00
ICD_Schizophrenia (F2*)	0.13	0.86
ICD_Substance_misuse (F1*)	0.16	0.98
ICD_Other	0.26	2.21
ICD_Total (Sum of all relevant ICD groups in the rows below)	0.23	1.76

Figure 6C: Ratio of scheduled NF2F versus F2F appointments by ICD-10 diagnostic group, in 2019 and 2020, in OHFT. Corresponds to Table 6B.

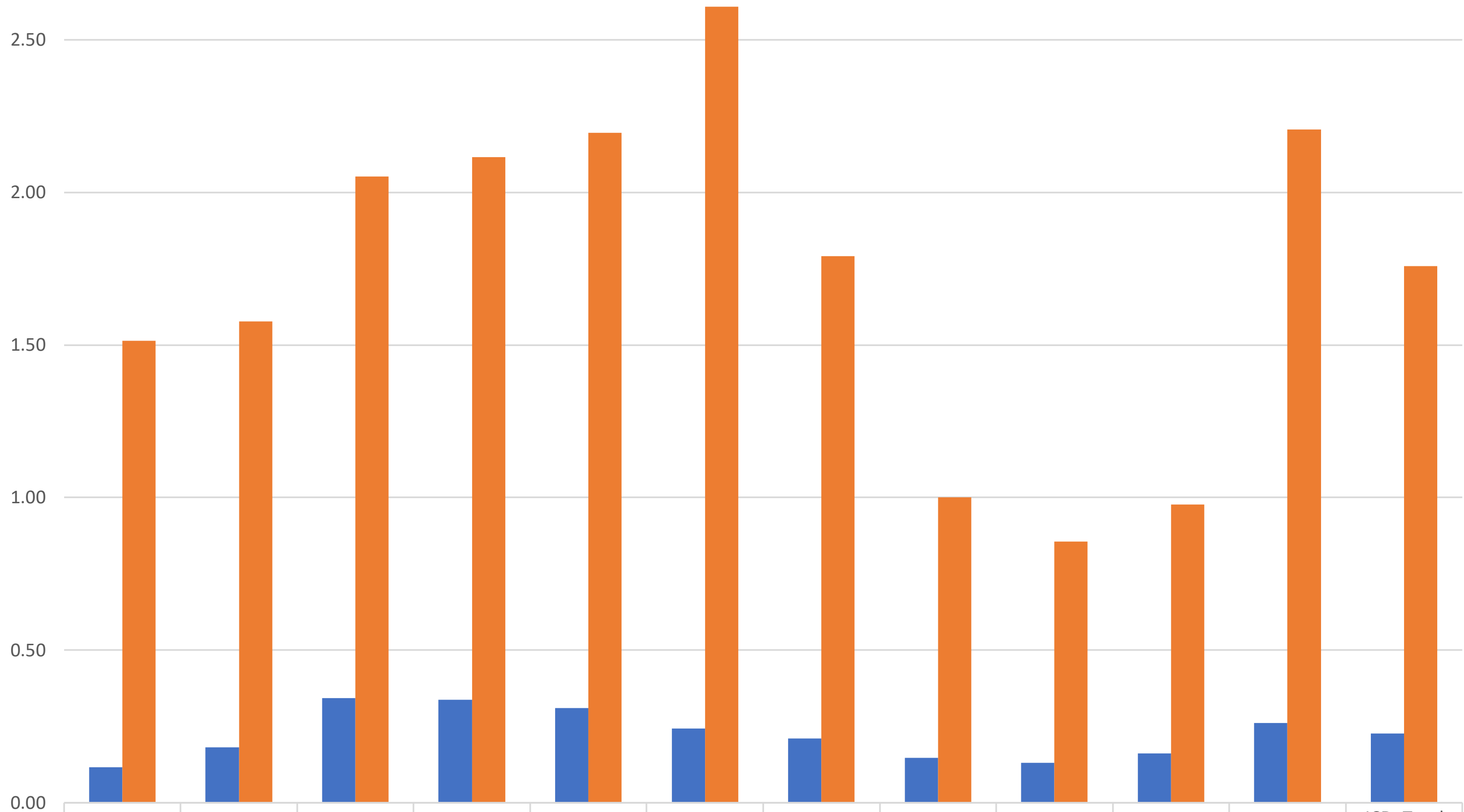

	ICD_Eating_Disorders (F50)	ICD_Affective_disorders (F3*)	ICD_Conduct_disorders (F90-F98)	ICD_Dementia (F00, F01, F02, F03)	ICD_Developmental_Disorders (F8*)	ICD_Mental_retardation (F7*)	ICD_Neurotic_Stress-related (F4*)	ICD_Personality_Disorders (F60-F61)	ICD_Schizophrenia (F2*)	ICD_Substance_misuse (F1*)	ICD_Other	ICD_Total (Sum of all relevant ICD groups in the rows below)
Ratio Scheduled NF2F to F2F 2019	0.12	0.18	0.34	0.34	0.31	0.24	0.21	0.15	0.13	0.16	0.26	0.23
Ratio Scheduled NF2F to F2F 2020	1.51	1.58	2.05	2.11	2.20	2.61	1.79	1.00	0.86	0.98	2.21	1.76

Figure 6D: Non-attended F2F appointments across diagnostic groups, as a percentage of scheduled F2F appointments, in 2020 in OHFT

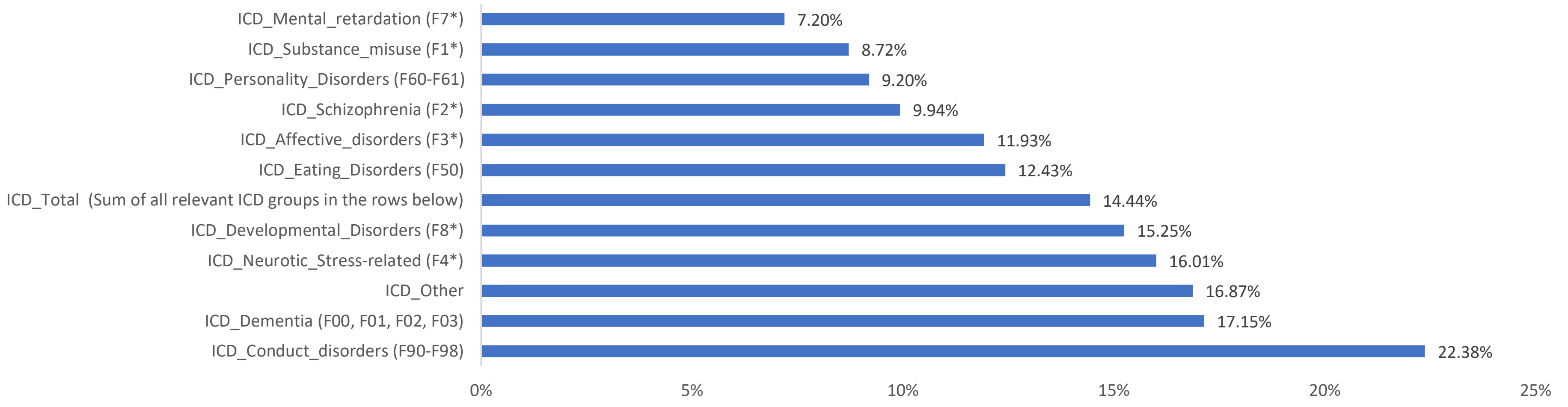

Figure 6E: Non-attended Non F2F appointments across diagnostic groups, as a percentage of scheduled Non F2F appointments, in 2020 in OHFT

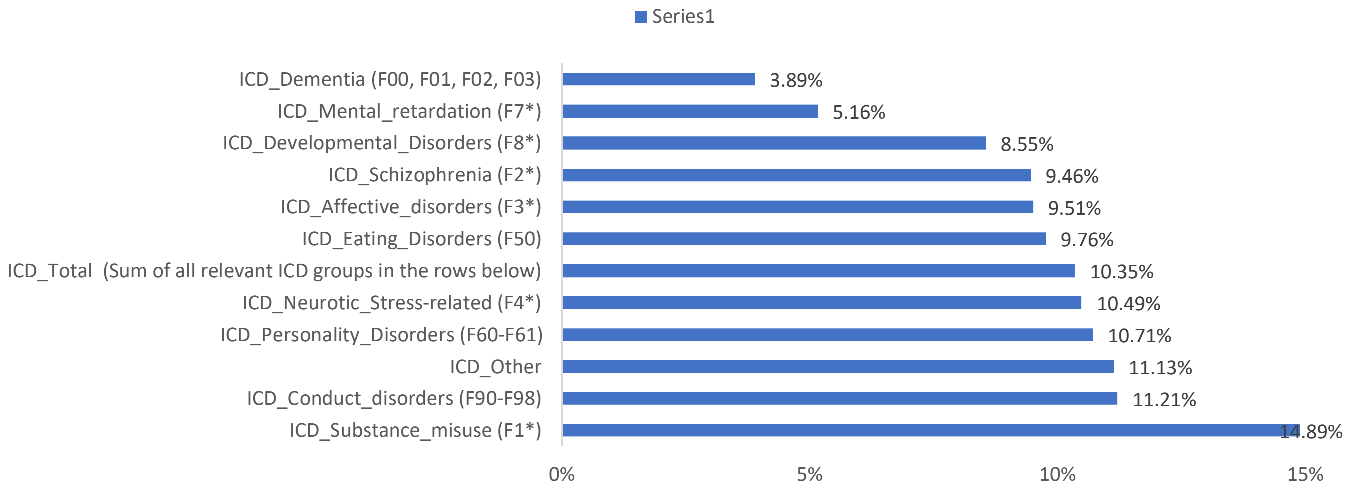

Figure 6F. Total CMHT activity in Southern Health NHS Foundation Trust (SHFT), in terms of F2F plus NF2F appointments, from 2019 to 2020. Total CMHT activity in SHFT, in terms of F2F plus NF2F appointments, was similar or increased in all teams from 2019 to 2020. Y-axis: number of appointments.

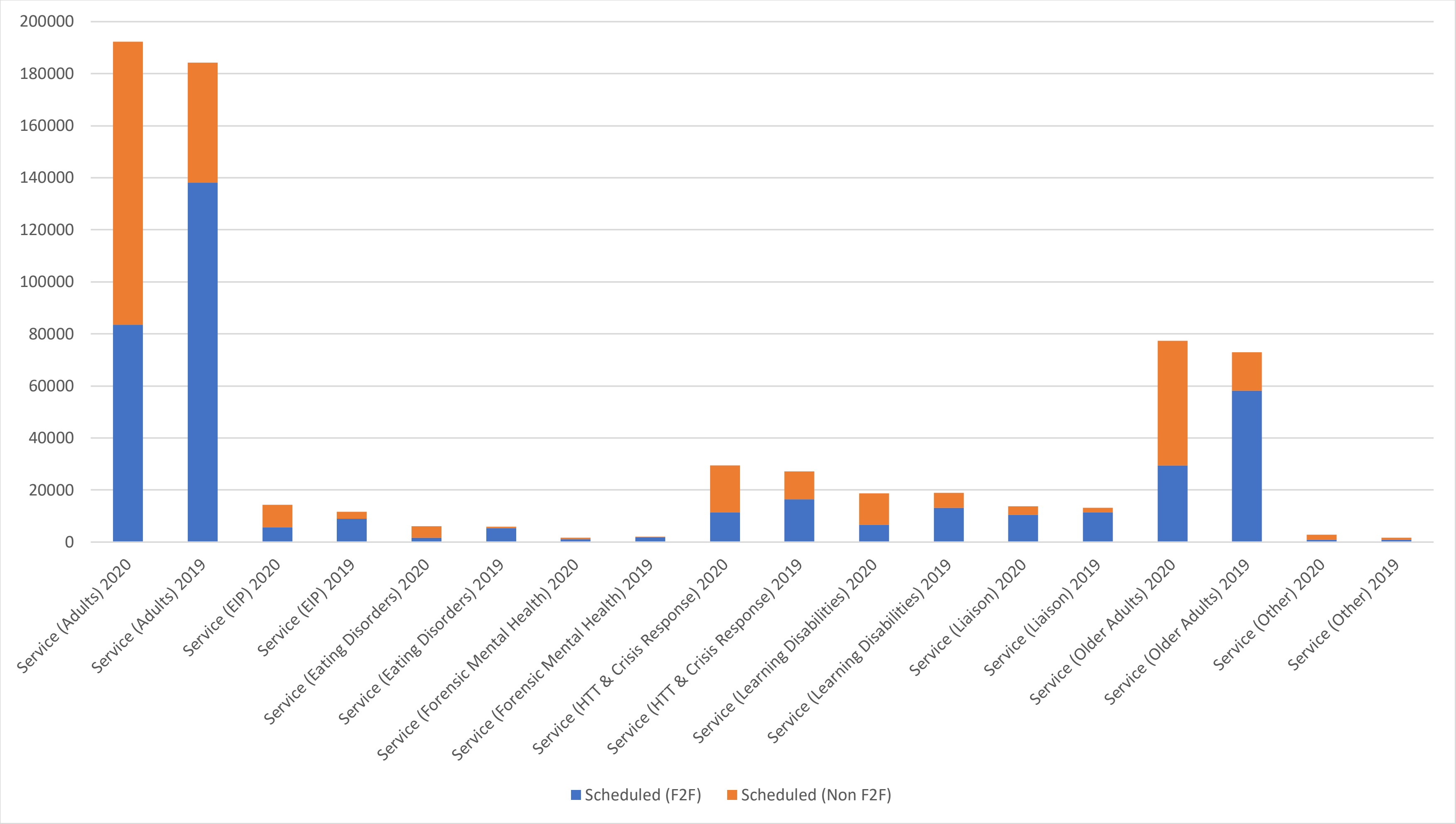

Table 6C: Ratio of scheduled NF2F versus F2F appointments by Clinical Team, in 2019 and 2020, in SHFT.

Clinical Team	Ratio Scheduled NF2F to F2F 2019	Ratio Scheduled NF2F to F2F 2020
Adults	0.33	1.30
EIP	0.30	1.47
Eating Disorders	0.10	2.53
Forensic mental health	0.14	0.48
HTT & Crisis response	0.65	1.56
Learning disabilities	0.43	1.79
Liaison	0.14	0.31
Older adults	0.25	1.62
Other	0.76	2.35

Hong JSW, *et al. Evid Based Ment Health* 2021;0:1–6. doi: 10.1136/ebmental-2021-300287

Figure 6G: Ratio of scheduled NF2F versus F2F appointments by Clinical Team, in 2019 and 2020, in SHFT. Corresponds to Table 6C.

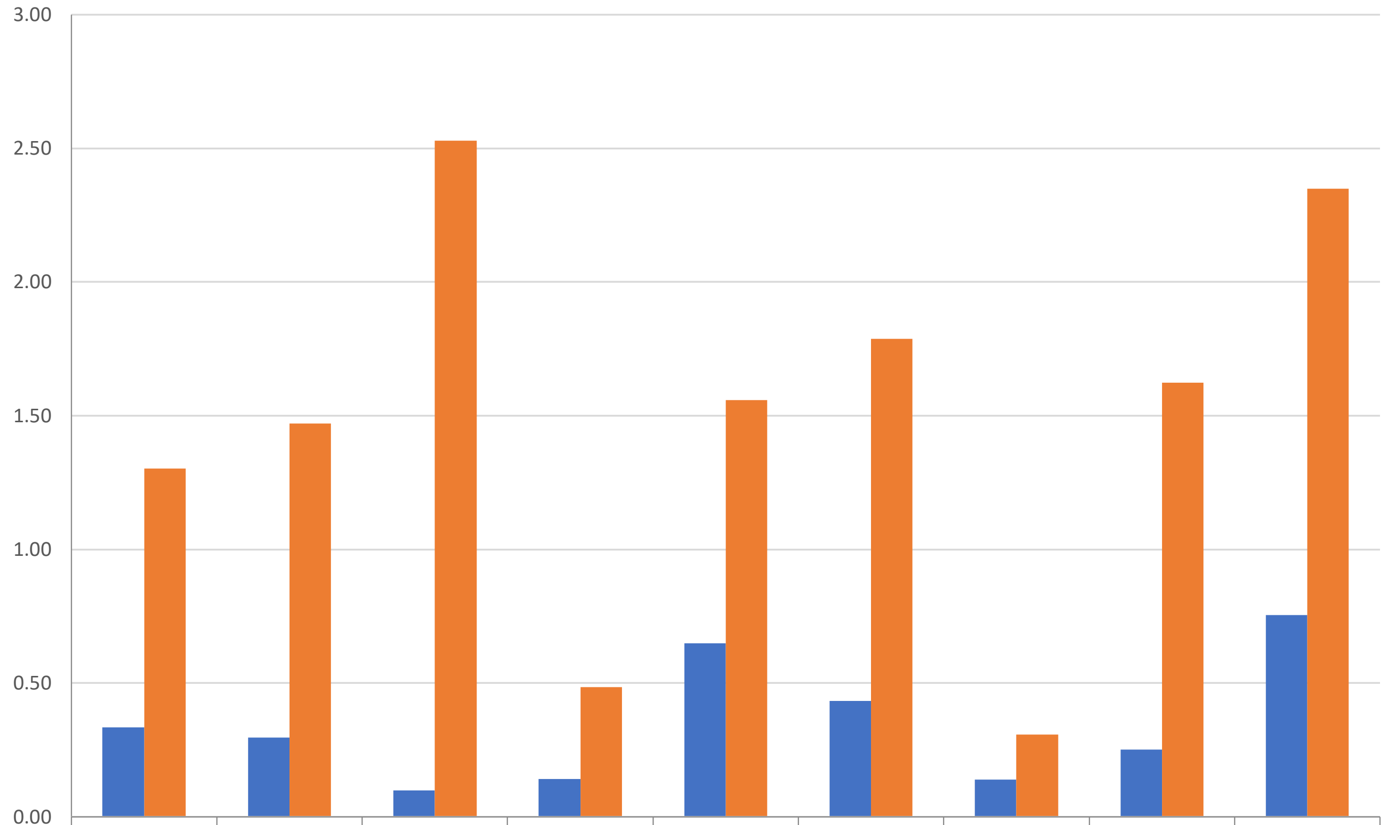

	Service (Adults)	Service (EIP)	Service (Eating Disorders)	Service (Forensic Mental Health)	Service (HTT & Crisis Response)	Service (Learning Disabilities)	Service (Liaison)	Service (Older Adults)	Service (Other)
Ratio Scheduled NF2F to F2F 2019	0.33	0.30	0.10	0.14	0.65	0.43	0.14	0.25	0.76
Ratio Scheduled NF2F to F2F 2020	1.30	1.47	2.53	0.48	1.56	1.79	0.31	1.62	2.35

Hong JSW, *et al. Evid Based Ment Health* 2021;0:1–6. doi: 10.1136/ebmental-2021-300287

Table 6D: Referrals per month in OHFT and SHFT in 2020.

Months of 2020	OHFT referrals	SHFT referrals
Jan	5,596	4,485
Feb	5,914	4,300
March	5,123	3,613
April	3,086	2,401
May	3,502	3,001
June	4,668	3,962
July	5,280	4,314
Aug	4,283	3,853
Sept	5,190	4,060

Figure 6H: Referrals per month in OHFT and SHFT in 2020. Corresponds to Table 6D.

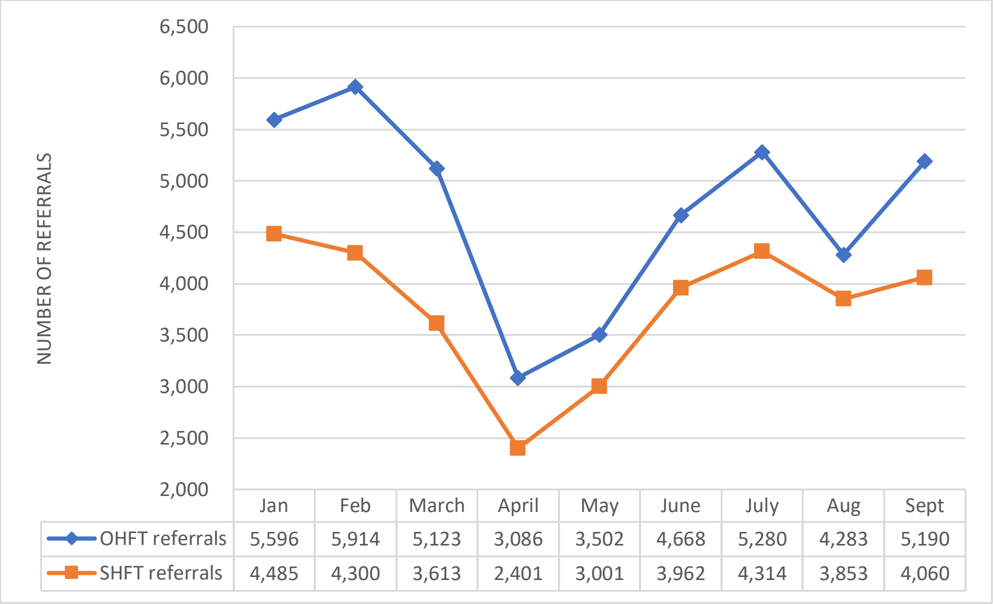

Table 6E: Monthly attendance rate at F2F (face-to-face/in-person) and Non-F2F (remote) appointments in 2020 in OHFT and SHFT.

Months of 2020	OHFT		SHFT	
	F2F	Non-F2F	F2F	Non-F2F
January	84.9%	93.2%	77.9%	87.1%
February	84.4%	92.4%	78.2%	87.1%
March	78.6%	92.2%	71.4%	85.9%
April	80.1%	91.4%	66.5%	84.3%
May	89.4%	90.0%	76.8%	83.0%
June	92.0%	89.3%	78.9%	82.8%
July	91.1%	88.7%	78.6%	82.3%
August	91.5%	87.9%	79.3%	83.1%
September	89.8%	87.9%	79.1%	82.6%
Monthly average (May to September 2020)	90.8%	88.8%	78.6%	82.7%

Table 6F: Non-attendance rates of F2F (face-to-face) and non-F2F consultations by ICD diagnosis in the year 2020 at Oxford Health NHS Foundation Trust. Non-attendance rate (%) = (Number not attended / scheduled appointments) x 100%

	F2F non-attendance rate (%)	Non-F2F non-attendance rate (%)	Ratio of F2F to Non-F2F non-attendance rate
ICD_Mental_retardation (F7*)	7.2	5.2	1.4
ICD_Substance_misuse (F1*)	8.7	14.9	0.6
ICD_personality_disorders (F60-F61)	9.2	10.7	0.9
ICD_Schizophrenia (F2*)	9.9	9.5	1.0
ICD_Affective_disorders (F3*)	11.9	9.5	1.3
ICD_Eating_disorders (F50)	12.4	9.8	1.3
ICD_Total	14.4	10.4	1.4
ICD_Developmental_Disorders (F8*)	15.3	8.6	1.8
ICD_Neurotic_Stress-related (F4*)	16	10.5	1.5
ICD_Other	16.9	11.1	1.5
ICD_Dementia (F00-F03)	17.2	3.9	4.4
ICD_Conduct_disorders (F90-F98)	22.4	11.2	2.0

Figure 6J: No-show rates* for F2F (face-to-face) and non-F2F appointments in Oxford and Southern NHS Trusts in 2020.

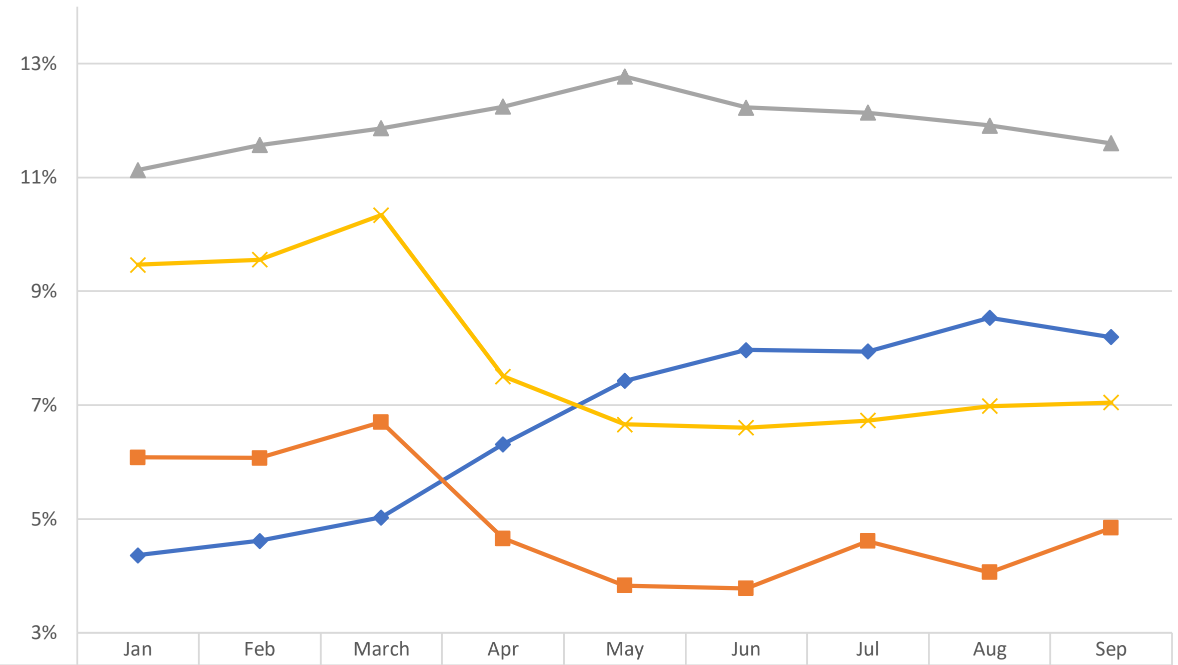

	Jan	Feb	March	Apr	May	Jun	Jul	Aug	Sep
Oxford, Non-F2F	4.4%	4.6%	5.0%	6.3%	7.4%	8.0%	7.9%	8.5%	8.2%
Oxford, F2F	6.1%	6.1%	6.7%	4.7%	3.8%	3.8%	4.6%	4.1%	4.8%
Southern, Non-F2F	11.1%	11.6%	11.9%	12.2%	12.8%	12.2%	12.1%	11.9%	11.6%
Southern, F2F	9.5%	9.6%	10.3%	7.5%	6.7%	6.6%	6.7%	7.0%	7.0%

***No-show rates were defined as follows: percentage of all scheduled appointments of the respective modality (F2F or Non-F2F) which were DNAs (Did Not Attend, aka. 'no-show', is recorded when a patient does not attend a scheduled appointment *and* there is no clear reason given by the patient or service for the non-attendance).**

No-show $\text{rate}_{\text{modality}}$ = (Number of $\text{DNA}_{\text{modality}}$ / Number of scheduled $\text{appointments}_{\text{modality}}$) x 100%

Section 7: HoNOS and Mental Health Care cluster analyses

Section 7.1: Cohort 1 Analyses

Cohort 1 Selection criteria (*Also described in Methods of the main text*)
Step 1 – Identify patients have at least 1 assessment in 2018, 2019 & 2020
Step 2 – If a patient has more than one assessment in a year, then the most recent assessment is included in the analysis.
Step 3 – identify patient moved between superclass in 2018, 2019 and 2020 and exclude them from the analysis

HoNOS Assessments	2018	2019	2020
Total Honos Assessment	10,720	10,872	9,538
Patients	998	998	998
Patient move superclass	206	206	206
Assessments in the analysis	792	792	792

Table 7A – Descriptive statistics for patients and HoNOS assessment per year in Cohort 1

The table below shows the number of patients in each superclass per year. Please note the patients move between clusters are excluded from the analysis.

SuperClass	2018	2019	2020
Non-psychotic	284	284	284
Organic	174	174	174
Psychotic	332	332	332
Var*	2	2	2
**Grand Total**	**792**	**792**	**792**

Table 7B – Number of patients per superclass per year in Cohort 1

***Var: Cluster 0 is called the 'variance' cluster, which codes patients who need mental health care, but cannot be classified into one of the other clusters. Cluster 9 is a 'blank cluster' and is not used.**

Please refer to the *Methods* section of the main text for a description of HoNOS scores, clusters, and superclasses.

Hong JSW, *et al. Evid Based Ment Health* 2021;0:1–6. doi: 10.1136/ebmental-2021-300287

Section 7.1A: HoNOS Scores

The descriptive statistics for each superclass for HoNOS scores are in the following table.

Year	N	Mean	Friedman Mean Rank	Std. Deviation	Min	Max
2018	284	12.64	2.07	5.86	1	36
2019	284	12.34	2.05	6.03	0	36
2020	284	11.88	1.87	8.02	0	99

Table 7C – The HoNOS Scores descriptive statistics for Non-Psychotic superclass

Year	N	Mean	Friedman Mean Rank	Std. Deviation	Min	Max
2018	332	11.84	2.16	6.44	0	37
2019	332	10.60	1.94	6.59	0	35
2020	332	10.36	1.90	6.12	0	31

Table 7D – The HoNOS Scores descriptive statistics for Psychotic superclass

Year	N	Mean	Friedman Mean Rank	Std. Deviation	Min	Max
2018	174	9.20	1.80	5.89	1	28
2019	174	10.17	1.98	6.21	1	27
2020	174	11.89	2.22	9.34	1	99

Table 7E – The HoNOS Scores descriptive statistics for Organic superclass

The results of applying Friedman statistical significance test on HoNOS scores for each superclass.

Year	N	Chi-Square	df	Asymp. Sig.
Non-Psychotic	284	7.729	2	0.210
Psychotic	332	14.772	2	<0.001
Organic	174	16.823	2	<0.001

Table 7F – The result of Friedman test for HoNOS scores for each superclass

Post-hoc Pairwise test

Post-hoc pairwise tests are commonly performed after a significant difference has been found when there are three or more levels of a factor. The Python package scikit-posthocs provides post hoc tests for pairwise multiple comparisons.

The result of applying Related-Sample Wilcoxon Signed-Rank post-hoc tests on Psychotic and Organic superclass are shown in Tables 7G and 7H respectively.

Years	Test Statistics	Std. Error	Srd. Test Statistic	Asymp. Sig. (2-sided test)	Adj. Sig.*
2018-2019	17652.00	1531.51	-3.610	0.001	0.003
2018-2020	19008.50	1630.34	-3.799	0.001	0.003
2019-2020	19584.00	1374.66	-0.370	0.711	1.000

Table 7G – Wilcoxon Post-hoc pairwise test result for Psychotic superclass

*** Significance values have been adjusted by the Bonferroni correction for multiple tests.**

Years	Test Statistics	Std. Error	Srd. Test Statistic	Asymp. Sig. (2-sided test)	Adj. Sig.*
2018-2019	6701.00	510.22	2.618	0.009	0.027
2018-2020	8018.50	537.44	4.243	<.001	0.003
2019-2020	6688.50	500.32	2.935	0.003	0.009

Table 7H – Wilcoxon Post-hoc pairwise test result for Organic superclass

*** Significance values have been adjusted by the Bonferroni correction for multiple tests.**

Section 7.1B: Clusters

The descriptive statistics for each superclass for HoNOS clusters are in the following three tables.

Year	N	Mean	Friedman Mean Rank	Std. Deviation	Min	Max
2018	284	4.58	2.05	1.865	1	8
2019	284	4.98	2.05	2.733	1	8
2020	284	4.51	1.89	2.148	1	8

Table 7I – The HoNOS Clusters descriptive statistics for Non-Psychotic superclass

Year	N	Mean	Friedman Mean Rank	Std. Deviation	Min	Max
2018	332	11.87	2.05	1.820	10	17
2019	332	11.58	1.91	2.227	10	17
2020	332	11.81	2.05	1.729	10	17

Table 7J – The HoNOS Clusters descriptive statistics for Psychotic superclass

Year	N	Mean	Friedman Mean Rank	Std. Deviation	Min	Max
2018	174	18.66	1.83	0.859	18	21
2019	174	18.49	1.93	2.119	18	21
2020	174	19.05	2.24.	0.987	18	21

Table 7K – The HoNOS Clusters descriptive statistics for Organic superclass

The results of applying Friedman statistical significance test on HoNOS clusters for each Superclass.

Year	N	Chi-Square	df	Asymp. Sig
Non-Psychotic	284	7.287	2	0.260
Psychotic	332	10.044	2	0.007
Organic	174	35.193	2	2.2-e08

Table 7L – The result of Friedman test for HoNOS clusters for each superclass

Post-hoc Pairwise test

The result of applying Related-Sample Wilcoxon Signed-Rank post-hoc tests on clusters for Psychotic and Organic superclasses are shown in Tables 7M and 7N below.

Years	Test Statistics	Std. Error	Srd. Test Statistic	Asymp. Sig. (2-sided test)	Adj. Sig.*
2018-2019	3480.00	447.09	-2.483	0.013	0.039
2018-2020	6372.00	583.60	-0.393	0.694	1.000
2019-2020	5563.50	457.94	1.677	0.094	0.282

Table 7M – Wilcoxon Post-hoc pairwise test result for Psychotic superclass

*** Significance values have been adjusted by the Bonferroni correction for multiple tests.**

Years	Test Statistics	Std. Error	Srd. Test Statistic	Asymp. Sig. (2-sided test)	Adj. Sig.*
2018-2019	1022.50	123.46	1.114	0.265	1.000
2018-2020	2689.00	196.08	5.452	0.001	0.003
2019-2020	1889.00	155.77	4.597	0.001	0.003

Table 7N – Wilcoxon Post-hoc pairwise test result for Organic superclass

*** Significance values have been adjusted by the Bonferroni correction for multiple tests.**

Section 7.2: Cohort 2 Analyses

Cohort 2 Selection Criteria (*Also described in Methods of the main text*)

Step 1 – Identify patients have at least 1 assessment in each year in 2019 and 2020

Step 2 – If a patient has more than one assessment in a year, then the most recent assessment is included in the analysis.

Step 3 – identify patient moved between superclass between 2019 and 2020 and exclude them from the analysis

HoNOS Assessments	2019	2020
Total Honos Assessment	10,872	9,538
Patients	2,712	2,712
Assessments for 2,712 patients	3,608	3,507
Patient move superclass	394	394
Assessments in the analysis	2318	2318

Table 7O - Descriptive statistics for patients and HoNOS assessment per year in Cohort 2

SuperClass	Patients
Non-psychotic	909
Organic	687
Psychotic	720
Var*	2
**Grand Total**	**2318**

Table 7P – Number of patients per superclass per year in Cohort 2

***Var: Cluster 0 is called the 'variance' cluster, which codes patients who need mental health care, but cannot be classified into one of the other clusters. Cluster 9 is a 'blank cluster' and is not used.**

Please refer to the *Methods* section of the main text for a description of HoNOS scores, clusters, and superclasses.

Section 7.2A: HoNOS Scores

The descriptive statistics for each superclass for HoNOS scores are in the following table.

Superclass	Year	N	Mean	Std. Deviation	Min	Max
Non-Psychotic	2019	909	12.182	6.835	0	99
	2020	909	11.617	6.826	0	99
Psychotic	2019	720	11.225	6.641	0	35
	2020	720	10.233	7.008	0	99
Organic	2019	687	9.395	6.761	0	99
	2020	687	10.444	8.367	1	99

Table 7Q – The HoNOS Scores descriptive statistics for all superclass

The Wilcoxon signed-rank test ranks for each superclass are in the following three tables.

		N	Mean Rank	Sum of Ranks
2020 - 2019	Negative Ranks	457[a]	424.24	193029.5
	Positive Ranks	377[b]	404.89	151835.5
	Ties	75[c]		
	Total	909		

a. 2020_ < 2019_
b. 2020_ > 2019_
c. 2020_ = 2019_

Table 7R – The HoNOS Scores Wilcoxon Ranks for Non-Psychotic superclass

Hong JSW, *et al. Evid Based Ment Health* 2021;0:1–6. doi: 10.1136/ebmental-2021-300287

Year		N	Mean Rank	Sum of Ranks
2020 - 2019	Negative Ranks	350[a]	321.25	112759
	Positive Ranks	261[b]	286.66	74819
	Ties	109[c]		
	Total	720		

a. 2020_ < 2019_
b. 2020_ > 2019_
c. 2020_ = 2019_

Table 7S – The HoNOS Scores Wilcoxon Ranks for Psychotic superclass

Year		N	Mean Rank	Sum of Ranks
2020 - 2019	Negative Ranks	248[a]	275.99	68721
	Positive Ranks	331[b]	302.26	100350
	Ties	108[c]		
	Total	687		

a. 2020_ < 2019_
b. 2020_ > 2019_
c. 2020_ = 2019_

Table 7T – The HoNOS Scores Wilcoxon Ranks for Organic superclass

The statistical significance result based on Wilcoxon Signed-Rank for each superclass is in the following table.

SuperClass	Z	Asymp. Sig. (2-tailed)
Non-Psychotic	-2.986[a]	0.003
Psychotic	-4.342[a]	<.001
Organic	-3.914[b]	<.001

[a] Based on positive ranks.

[b] Based on negative ranks.

Table 7U – The result of Wilcoxon Signed-Rank test for HoNOS scores for each superclass in Cohort 2.

Section 7.2B: Clusters

The descriptive statistics for each superclass for HoNOS clusters are in the following table.

Superclass	Year	N	Mean	Std. Deviation	Min	Max
Non-Psychotic	2019	909	4.5	1.959	1	8
	2020	909	4.41	2.098	1	8
Psychotic	2019	720	11.75	1.844	10	17
	2020	720	11.73	1.656	10	17
Organic	2019	687	18.64	0.865	18	21
	2020	687	18.86	0.909	18	21

Table 7V – The HoNOS Cluster descriptive statistics for all superclass

The Wilcoxon signed-rank test ranks for each superclass are in the following three tables.

		N	Mean Rank	Sum of Ranks
2020 - 2019	Negative Ranks	324[a]	277.58	89382
	Positive Ranks	260[b]	306.62	79108
	Ties	325[c]		
	Total	909		

a. 2020_ < 2019_
b. 2020_ > 2019_
c. 2020_ = 2019_

Table 7W – The HoNOS Cluster Wilcoxon Ranks for Non-Psychotic superclass

		N	Mean Rank	Sum of Ranks
2020 - 2019	Negative Ranks	129[a]	161.66	20854
	Positive Ranks	160[b]	132.55	21341
	Ties	431[c]		
	Total	720		

a. 2020_ < 2019_
b. 2020_ > 2019_
c. 2020_ = 2019_

Table 7X – The HoNOS Cluster Wilcoxon Ranks for Psychotic superclass

		N	Mean Rank	Sum of Ranks
2020 - 2019	Negative Ranks	70[a]	131.81	9227
	Positive Ranks	191[b]	131.39	25226
	Ties	426[c]		
	Total	687		

a. 2020_ < 2019_
b. 2020_ > 2019_
c. 2020_ = 2019_

Table 7Y – The HoNOS Cluster Wilcoxon Ranks for Organic superclass

The statistical significance result based on Wilcoxon Signed-Rank for each superclass is in the following table.

SuperClass	Z	Asymp. Sig. (2-tailed)
Non-Psychotic	-1.283[a]	0.199
Psychotic	-.175[b]	0.861
Organic	-6.930[b]	<.001

[a] Based on positive ranks.

[b] Based on negative ranks.

Table 7Z – The result of Wilcoxon Signed-Rank test for HoNOS cluster for each superclass in Cohort 2.

Section 8: Inpatient service data

Table 8A: Inpatient service data (turnover) in 2018, 2019, and 2020 in Oxford Health NHS Foundation Trust (OHFT) and Southern Health NHS Foundation Trust (SHFT).

	OHFT	SHFT
**Inpatients (in hospital at least once during the specified period)**		
**2018**	1,335	1,855
**2019**	1,370	1,860
**2020**	1,301	1,870
**Number of new admissions as inpatients**		
**2018**	1,077	1,626
**2019**	1,088	1,698
**2020**	1,033	1,744
**Number of discharged inpatients**		
**2018**	1,075	1,590
**2019**	1,106	1,696
**2020**	1,022	1,839

Table 8B: Number of occupied beds per day in OHFT and SHFT in 2018, 2019, and 2020.

	OHFT	SHFT
**Inpatients (number of occupied beds per day)**		
**2018**	117,712	135,849
**2019**	115,144	131,461
**2020**	106,574	113,252

Hong JSW, *et al. Evid Based Ment Health* 2021;0:1–6. doi: 10.1136/ebmental-2021-300287

Table 8C: Number of distinct patients with a hospital stay per month in OHFT and SHFT in 2020.

	OHFT patients	SHFT patients
**Jan**	514	673
**Feb**	536	627
**March**	528	634
**Apr**	470	522
**May**	483	535
**June**	492	574
**July**	494	602
**Aug**	497	559
**Sept**	494	573

Figure 8A: Number of inpatients (distinct patients with a hospital stay) per month in OHFT and SHFT in 2020. Corresponds to Table 8C.

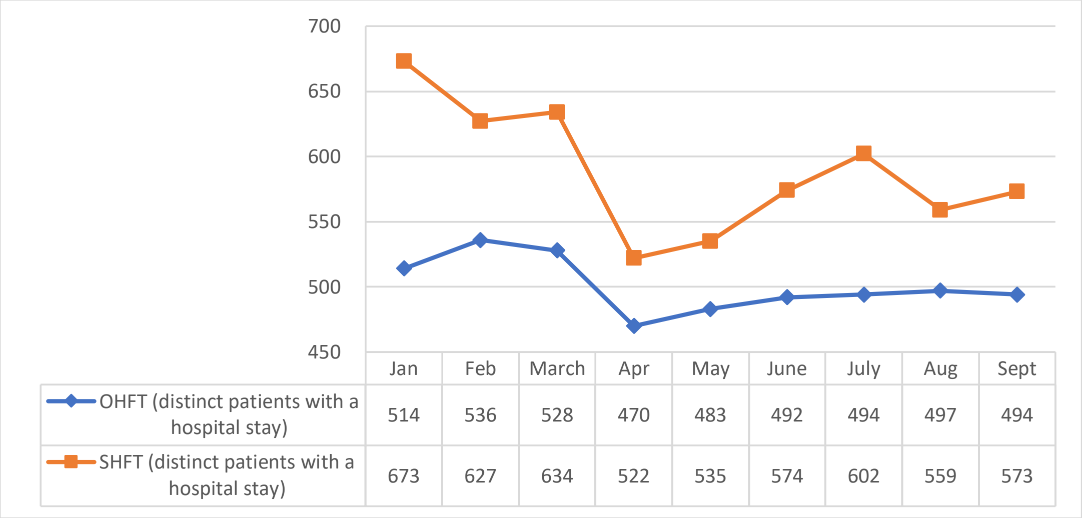

Hong JSW, *et al. Evid Based Ment Health* 2021;0:1–6. doi: 10.1136/ebmental-2021-300287

References

1. S. Rathod, S. Pallikadavath, A.H. Young, L. Graves, M.M. Rahman, A. Brooks, et al. Psychological impact of COVID-19 pandemic: protocol and results of first three weeks from an international cross-section survey – focus on health professionals. J Affecffect Disord Rep 2020;100005. doi:10.1016/j.jadr.2020.100005.

2. Phiri, P., Ramakrishnan, R., Rathod, S., Elliot, K., Thayanandan, T., Sandle, N., Haque, N., Chau, S.W., Wong, O.W.H., Chan, SSM., Wong, E.K.Y., Raymont, V., Au-Yeung, S.Y., KingdonN, D., Delanerolle, G. (2021). An evaluation of the mental health impact of SARS-CoV-2 on patients, general public and healthcare professionals: A systematic review and meta-analysis EClinicalMedicine ISSN: Vol: 34, Page: 100806 DOI10.1016/j.eclinm.2021.100806

3. Chau, S.W.H., Wong, O.W.H., Ramakrishnan, R, Chan, S.S.M., Wong, E.K.Y., Li, P.Y.T., Raymont, V., Elliot, K., Rathod, S., Delanerolle, G., Phiri, P. History for some or lesson for all? A systematic review and meta-analysis on the immediate and long-term mental health impact of the 2002–2003 Severe Acute Respiratory Syndrome (SARS) outbreak. BMC Public Health 21, 670 (2021). https://doi.org/10.1186/s12889-021-10701-3

4. American Psychiatric Association. History of Telepsychiatry. 2021, Last accessed 20 May 2021. Available at: https://www.psychiatry.org/psychiatrists/practice/telepsychiatry/toolkit/history-of-telepsychiatry.
